# Benign prostatic hyperplasia: An overview of existing treatment

**DOI:** 10.4103/0253-7613.75657

**Published:** 2011-02

**Authors:** Neelima Dhingra, Deepak Bhagwat

**Affiliations:** Pharmaceutical Chemistry Division, University Institute of Pharmaceutical Sciences, Panjab University, Chandigarh - 160 014, India; 1 Amar Shaheed Baba Ajit Singh Jujhar Singh Memorial College of Pharmacy, Bela (Ropar) - 140 111, Punjab, India

**Keywords:** Benign prostatic hyperplasia, prostate, treatment, α1-adrenergic antagonist, 5α-reductase inhibitors

## Abstract

Benign prostatic hyperplasia (BPH) is the most common condition in aging men, associated with lower urinary tract symptoms (LUTS). A better understanding of the prostate physiology, function, and pathogenesis has led to the development of promising agents, useful in the management of LUTS in men. The specific approach used to treat BPH depends upon number of factors like age, prostrate size, weight, prostate-specific antigen level, and severity of the symptoms. 5α-reductase inhibitors decrease the production of dihydrotestosterone within the prostate, which results in decreased prostate volume, increased peak urinary flow rate, improvement of symptoms, decreased risk of acute urinary retention, and need for surgical intervention. α_1_-adrenergic receptor (α_1_-AR) antagonists decrease LUTS and increase urinary flow rates in men with symptomatic BPH, but do not reduce the long-term risk of urinary retention or need for surgical intervention. Clinical efficacy of either 5α-reductase inhibitor or α_1_-AR antagonist has been further improved by using combination therapy; however, long-term outcomes are still awaited. Many more potential new therapies are under development that may improve the treatment of BPH. This article gives a brief account of rationale and efficacy of different treatment options presently available in the management of BPH.

## Introduction

**Figure 1:**
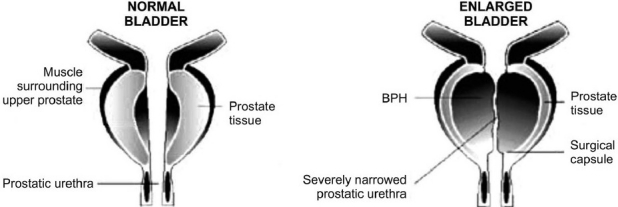
Normal and enlarged prostate

Benign prostatic hyperplasia (BPH) is the nonmalignant enlargement of the prostate gland. It refers to stromal and glandular epithelial hyperplasia that occurs in the periurethral transition zone of the prostate that surrounds the urethra [Figure 1]. BPH clinically manifest as lower urinary tract symptoms (LUTS) consisting of irritative (urgency, frequency, nocturia) and obstructive symptoms (hesitancy, a weak and interrupted urinary stream, straining to initiate urination, a sensation of incomplete bladder emptying).[1] Prolonged obstructions may eventually lead to acute urinary retention (AUR), recurrent urinary tract infection (UTI), hematuria, bladder calculi, and renal insufficiency.[2] The prevalence of LUTS due to BPH increases with increasing age. Moderate to severe symptoms occur in 40 and 80% of men after the age 60 and by 80 years, respectively. Nearly all men develop microscopic BPH by the age of 90 years.[3] It is also described as quality of life disorder, affecting man’s ability to initiate or terminate urine flow stream (the symptoms interfere with the normal activities), and reduces the feeling of well being. The causes of BPH are not fully known, but the overgrowth of smooth muscle tissue and glandular epithelial tissue is attributed to a number of different causes such as aging, late activation of cell growth, genetic factors, and hormonal changes.[1,4,5]

## Treatment options

**Figure 2:**
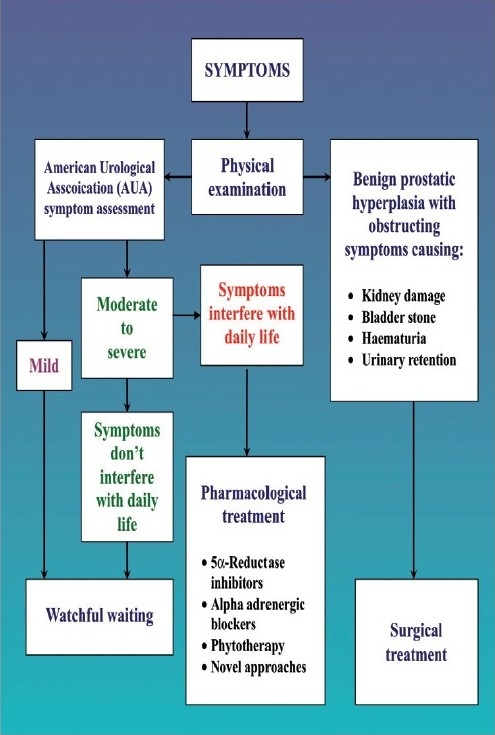
A schematic presentation of management of benign prostatic hypertrophy

A more profound knowledge of the pathogenesis, the natural history, and risk of the progression enabled more differentiated therapy of elderly men with BPH. The specific approach used to treat BPH depends upon a number of factors like age, prostate size, weight, prostate-specific antigen level, and severity of the symptoms [Figure 2].[6,7]

The options are as follows:

1. Watchful waiting
2. Pharmacological treatment
3. Surgical treatment

### 

#### 1. Watchful waiting

As long as the symptoms are mild and not causing any change in the day-to-day activities, wait and watch approach with regular check-up is recommended. However, if the symptoms are troublesome, pharmacological treatment is recommended. And with potentially serious symptoms, surgical interventions are considered.[8]

#### 2. Pharmacological treatment

**Figure 3:**
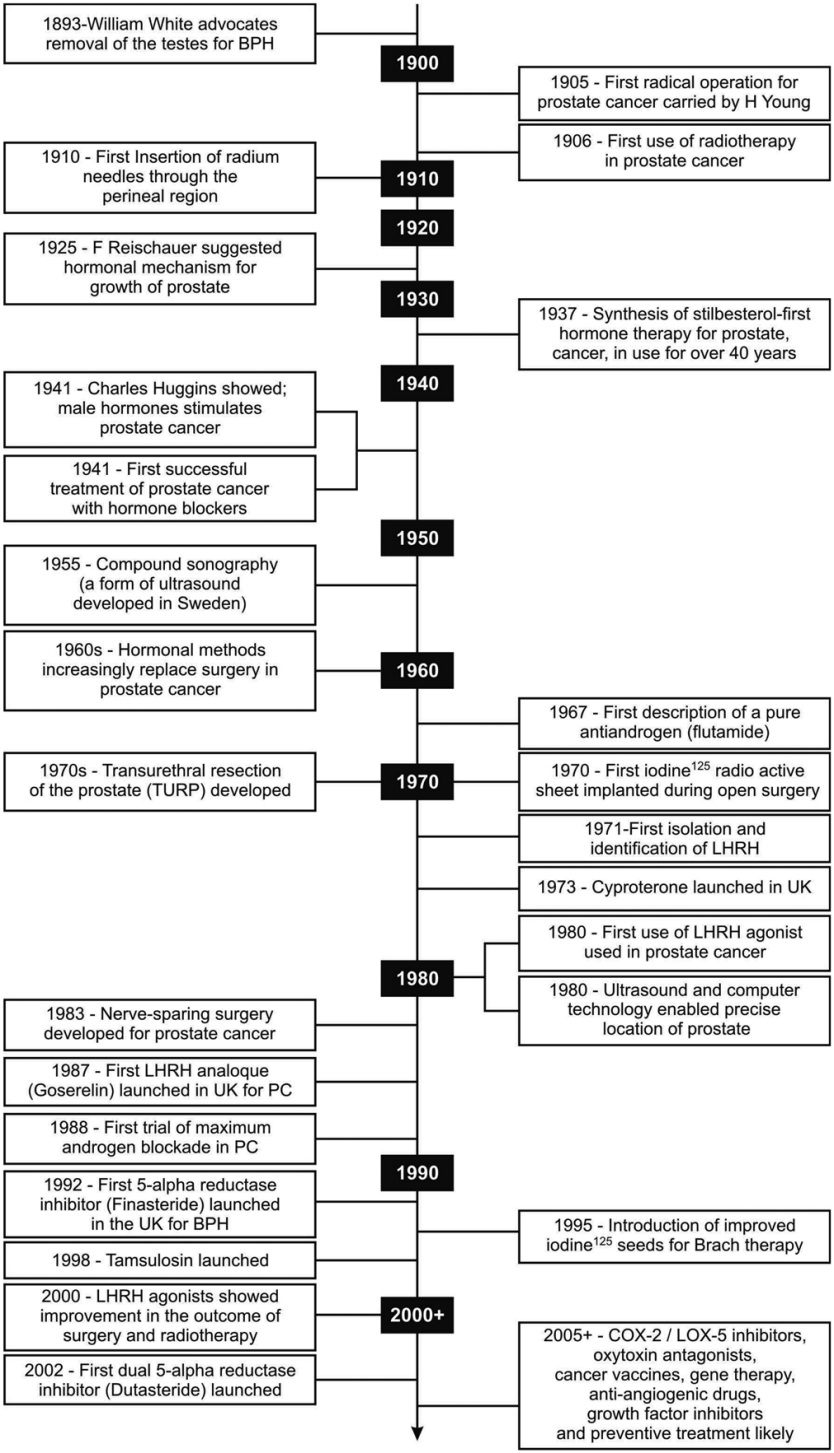
Milestones in understanding and treatment of BPH

The clinical manifestations of BPH are caused by increased resistance to the flow of urine through the bladder neck and compressed prostatic urethra. Medical treatments have been devised to decrease the size of the prostate, and there by decrease the resistance to urinary flow. Milestones in understanding and treatment have been shown in Figure 3.

In 1925, it was suggested that hormones regulate the growth of the prostate. In 1937, the first medicine, stilbestrol was synthesized for prostate and remained in use for 40 years, despite its several side effects. Further research led to the development of new medicines with improved properties, such as antiandrogens and hormones. Identifying the role of luteinizing hormone releasing hormone (LHRH) in regulating sex hormone in men and women was a landmark research that led to the development of LHRH agonists. Parallel developments in surgery, medical instrumentation, and radiation science also served as successful treatment for BPH.[9]

The aim of treatment of BPH is to improve symptoms, relief obstruction, improve bladder emptying, prevent UTIs, and avoid renal insult. A number of strategies are available but great strides in the development of antiandrogen (androgen deprivation therapy) and α-adrenergic blockers have fueled this evolution [Table 1].

**Table 1:** Culture results of ocular specimens

*Drug*	*Dosage*	*Mechanism*	*Side effects*
Finasteride	5 mg once daily	5α-reductase inhibitor	Impotence, decreased libido, decreased semen quantity at ejaculation, decreased semen prostate specific antigen, gynecomastia (rare)
Dutasteride	0.5 mg once daily	5α-reductase inhibitor	Impotence, decreased libido, decreased semen quantity at ejaculation, decreased semen prostate specific antigen, gynecomastia (rare)
Terazosin	1 mg once daily to start; may increase up to 10 mg/day	α1-adrenergic receptor antagonist	Asthenia, hypotension, dizziness, somnolence
Doxazosin	1 mg once daily to start; may increase up to 8 mg once daily	1-adrenergic receptor antagonist	Orthostatic hypotension, fatigue, dyspnea
Tamsulosin	0.4/0.8 mg once daily	α1-adrenergic receptor antagonist	Dizziness, rhinitis, abnormal ejaculation
Alfuzosin	2.5 mg t.i.d./5 mg b.i.d./10 mg once daily	α1-adrenergic receptor antagonist	Fatigue, edema, rhinitis, headache, upper respiratory tract infection
Saw palmetto	160 mg twice daily	Mixed	Aggravate chronic gastrointestinal disease such as peptic ulcer

## Androgen deprivation therapy

**Figure 4:**
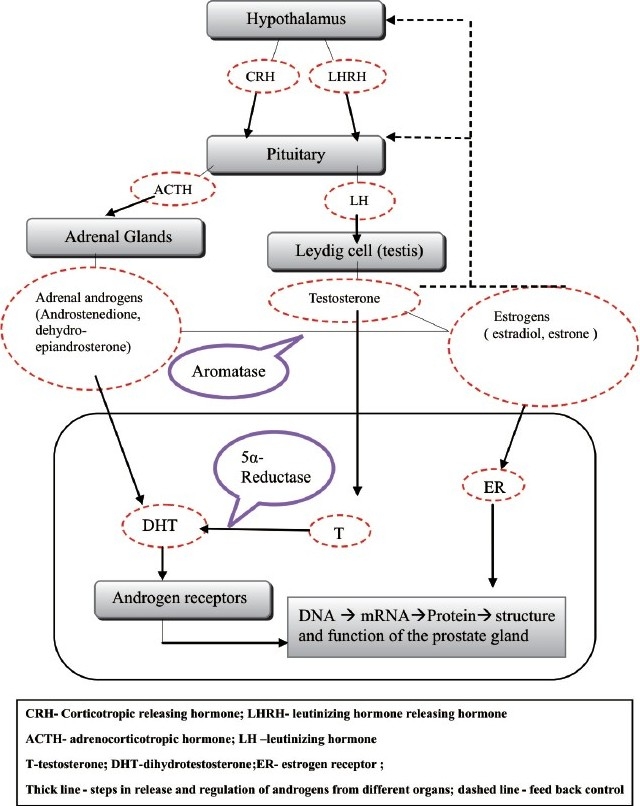
Androgen deprivation therapy

The biological basis of androgen ablation therapy lies in the observation that the embryonic development of the prostate is dependent on the androgen dihydrotestosterone (DHT) [Figure 4]. Furthermore, castration in men before puberty resulted into regression of prostatic enlargement.[10] Androgen deprivation causes the reduction in prostatic volume that is believed to reduce the static component of BPH.[11] Reversible androgen deprivation can be achieved by the use of progestational agents (hydroxyprogesterone acetate,[12] megesterone[13]) capable of decreasing serum testosterone levels by inhibiting the release of luteinizing hormone. Suppression of sex steroid production on the basis of desensitization and down regulation of pituitary gonadotropin releasing hormone (GnRH) receptor by agonistic GnRH analogues[14] (nafarelin acetate, leuprolide) resulting in the blockage of gonadotropin release from the anterior pituitary gland is a well-established approach in the treatment of BPH.[15,16] Furthermore, antiandrogens like cyproterone acetate[17] and flutamide[18] competitively inhibit the ligand (DHT) binding to the androgen receptor and are used therapeutically in BPH. Several lines of evidence indicate the role of estrogen along with androgen in BPH. Estrogens are mainly produced in men by aromatase activity by the peripheral conversion of testicular and adrenal androgen into estradiol. The estrogenic effect presumably includes its stromal and epithelial interaction that regulates the proliferative activity of the prostate and alteration in the sensitivity of the prostate toward androgens.[19] Aromatase inhibitors like atermestone[20] and abiraterone[21] that block the peripheral conversion have found application in pharmacological treatment of BPH.

Though the androgen deprivation therapy has proved to be an effective treatment, their use was restricted because of associated side effects such as erectile dysfunction and loss of libido.[22,23] Therefore, search for the new drugs with more efficacies, selectivity, and relative broader therapeutic index was being pursued, and continued accrual resulted in the development of 5α-reductase inhibitors.

### 

#### 5α-Reductase inhibitors

Steroidal 5α-reductase is a NADPH-dependent enzyme that catalyzes the irreversible conversion of 4-en-3-oxo-steroid, that is, testosterone (T), the major circulating androgen in male adults, to the corresponding 5α-H-3-oxo-steroid, that is, DHT. Two isozymes of 5α-reductase have been cloned, expressed, and characterized on the basis of differences in chromosomal localization, tissue expression pattern, and biochemical properties.[24] Within the prostate, locally produced DHT acts in a paracrine fashion to stimulate growth. However, excessive production of DHT is the cause of major androgen- related disorders such as prostate cancer, acne, female hirsutism, and BPH.[1] Therefore, inhibitors of androgen action by 5α-reductase is a logical treatment of 5α-reductase activity disorder, that is, BPH. These agents suppress the DHT concentration by blocking the enzyme, resulting in shrinkage in the size of prostate, increased peak urinary flow rates, and ultimately providing relief from the symptoms related to the static mechanical obstruction caused by BPH.[25] Furthermore, the rationale for use of 5α-reductase inhibitors is rooted in the observation that these are more specific to DHT androgens action without affecting or lowering T level, thus capable of decreasing long-term side effect of castration due to loss of T without compromising the efficacy of hormonal therapy.[26,27] Finasteride and dutasteride are commercially available 5α-reductase inhibitors currently being used in the treatment of BPH.

#### Finasteride

**Figure 5:**
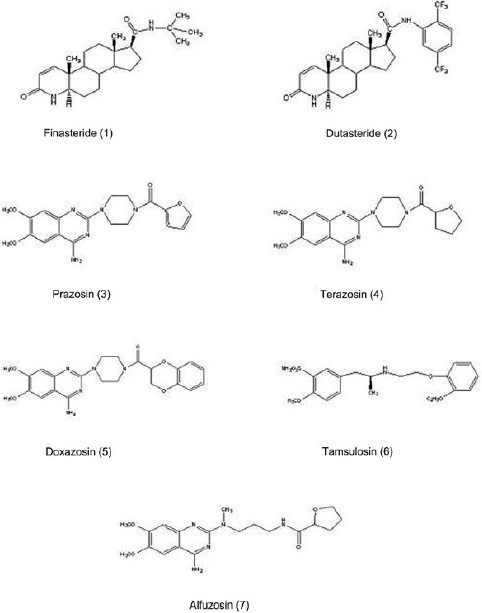
Structures of some potent reported compounds

Finasteride (MK-906) [Figure 5] synthesized in 1984, is chemically 17β-(N-tert-butyl-carbamoyl)-4-aza-5α-androst-1-en-3-one . It was the first 5α-reductase inhibitor approved in the United States in 1992 for the treatment of BPH.[28] Finasteride is a competitive inhibitor of 5α-reductase type 2 with 10-fold high affinity than type 1 and forms a stable complex with enzyme. It has been reported that at clinical doses of 5 mg/day in human beings, it decreases the prostate DHT level by 70 to 90%, thus resulting in decreased prostate volume or size and improved urinary flow rate.[29,30] It has neither androgenic, antiandrogenic, other hormone related properties, nor it interferes with the binding of T or DHT to the androgen receptor.[31] The investigators found significant improvement in finasteride-treated groups in term of increased flow rates and decreased prostate-specific antigen level. The most commonly reported side effects on finasteride long-term usage are decreased libido, ejaculatory dysfunction, or impotence, while some of the patients showed rashes and breast enlargement.[7]

#### Dutasteride

Dutasteride belongs to class of 4-aza-steroids and chemical name is 17-N-{2, 5-bis (trifluoromethyl) phenyl)}-3-oxo-4-aza-5α-androst-1-ene-17-carboxamide [Figure 5].[30] It was approved by US FDA in 2002 for the symptomatic treatment of BPH. Unlike finasteride, dutasteride has been reported to be a nonselective competitive inhibitor of both 5α-reductase type 1 and 5α-reductase type 2 isozymes. At clinical dose of 0.5 mg/day, it has been shown to decrease DHT levels >90%, by forming a stable complex with a slow rate of dissociation constant. Dutasteride was found to improve urinary flow rate, decrease the risk of AUR and need for surgery by reducing the size of enlarged prostate.[32–34] Improved efficacy of dutasteride (0.5 mg/day) over finasteride (5 mg/day) in terms of symptom score, maximal urinary flow rate, and quality of life has been reported in recently published article by Kumar *et al*.[30]

## Alpha adrenergic blockers

The rationale for using α-adrenergic blockers is based on the fact that noradrenaline acts at α_1_-adrenergic receptors (α _1_-AR) in the neck and sphincter of the urinary bladder to promote contraction and urinary retention, and control the smooth muscles in the prostate capsule and prostate urethra.[35,36] Therefore, selective α_1_-AR antagonists relieve the obstruction due to dynamic component by relaxing the smooth muscle in and around the prostate and bladder neck without affecting the detrusor muscle of the bladder wall. Molecular studies have further identified three subtypes of the α_1_-AR(α_1A_, α_1B_, and α_1D_). Their relative distribution and concentration in the prostate, bladder, neck, brain, and vascular smooth muscle have been exploited to develop uroselective α_1_-adrenergic antagonists and reduce side effects. The α_1B_ subtype is predominant in blood vessels, whereas α_1A_ is predominant in prostate.[37] Prazosin was the first selective α_1_-AR antagonist investigated for BPH treatment [Figure 5].[38] Prazosin contains a piperazinyl quinazoline nucleus and is selective α_1_-adrenergic antagonist, with affinity 1000-fold greater than that for α_2_-receptor. The adverse effects related to prazosin were postural hypotension along with stuffy nose, headache, and retrograde ejaculation on continuous use for a long period.[39]

The advent of selective α_1_-drugs, terazosin and doxazosin [Figure 5], the structural analog of prazosin, originally developed as antihypertensive agents.[39] Terazosin and doxazosin with long half life are given once a day with dose titration over 1 to 2 weeks due to first dose syncope to a maximum of 10 mg for terazosin and 8 mg for doxazosin.[7] Lepor reported significant increase in average urine flow rates without affecting voided or residual volume. The clinical efficacy and safety of terazosin and doxazosin documented in several studies have shown that terazosin therapy does not affect blood pressure control in patients receiving concurrent antihypertensive medication, whereas mild to moderate adverse events like fatigue dizziness has been observed with doxazosin.[38] Currently, tamsulosin and alfuzosin are the most widely prescribed medications as selective α_1_-AR antagonists for the LUTS associated with BPH.

### 

#### Tamsulosin

Tamsulosin hydrochloride is a competitive antagonist of α_1_-AR with the chemical name (-)-(R)-5-[2-[[2-(o-Ethoxyphenoxy) ethyl] amino] propyl] -2-methoxybenzenesulfonamide [Figure 5].[1] It was the third uroselective α_1_-AR antagonist with 10-fold more selectivity for α_1A_-receptor subtype compared with α_1B_-receptor subtype, approved for use in the treatment of symptomatic BPH.[38] It is well absorbed orally with half-life of 5 to 10 hours and extensively metabolized by the cytochrome P450 system.[40] A significant reduction in urinary flow has been observed after single dose (0.4 or 0.8 mg) administration of tamsulosin as compared with placebo.[38] Tamsulosin have minimal cardiovascular effects and the risk of dizziness is less as compare with doxazosin and prazosin.[41] The drug also demonstrated a lower probability of orthostatic hypotension, but a higher rate of ejaculatory dysfunction (10%), and does not appear to cause erectile dysfunction or reduced sexual drive.[41]

#### Alfuzosin

Alfuzosin is a quinozoline-based α_1_-AR antagonist with similar affinity for all α_1_receptor subtypes [Figure 5].[39,7] It is available in an immediate (2.5 mg t.i.d.), sustained (5 mg b.i.d.), and extended release formulation (10 mg/day) to improve compliance.[7] According to AUA guidelines, alfuzosin has comparable clinical efficacy with tamsulosin and the other approved alpha blockers and does not cause ejaculatory dysfunction.[38]

## Combination therapy

The scientific rationale for combining 5α-reductase inhibitors and α_1_-AR antagonists is based on their different and complementary modes of action, helpful to manage static and dynamic component in patients with an enlarged prostate gland having symptoms of bladder outlet obstruction. The rationale for this recommendation is a rapid relief of symptoms by the α_1_-AR antagonists, without targeting the underlying disease process and a mid or more sustained relief of symptoms by the 5α-reductase inhibitors.[41] The efficacy and safety of the treatment with different combinations versus treatment with either agent alone has been investigated by different groups in large mulitcentral trials.[2,7]

The Veterans Affairs Cooperative Study and Prospective European Doxazosin and Combination therapy evaluated the combination of finasteride with terazosin and doxazosin, respectively, for one year. Treatment with α_1_-AR antagonists alone or combination therapy significantly improved the symptom score and flow rate compared with placebo or finasteride alone, but there was no significant difference observed for combination therapy over α _1_-AR antagonists alone. These trials were subsequently followed by Medical therapy for Prostate Symptoms, wherein combination of finasteride and doxazosin was studied over a period of 4.5 years. It was found that risk of AUR and need for BPH-related surgery were significantly lower in finasteride and combination therapy versus placebo, whereas none of these outcomes was reduced significantly with doxazosin alone.

The combination of avodart and tamsulosin (CombAT)[1,30] study is underway to further examine the role of combination (dutasteride and tamsulosin) over the α_1_-AR antagonists (tamsulosin). It would be a major step in assessing the combination therapy and the findings will assist in making treatment decision. These studies demonstrated a higher incidence of impotence with combination therapy compared with 5α-reductase inhibitors, in addition to higher incidence of α_1_-AR antagonists-mediated dizziness, hypotension.[2] Cost-effectiveness studies by Nickel suggest that the combination therapy is more suitable for men at high risk for BPH progression (i.e., with high symptom score, large prostate volume and low q_max_ value) who are able to tolerate the increased side effects.

## Phytochemical agents

The use of plant-derived nonnutritive compounds with protective or disease-preventive properties for urinary symptoms with BPH has gained widespread interest, probably due to perceived reduction in side effects, and desire to maintain control over their treatment.[42,43] However, the use of these phytochemicals is controversial as most of the studies have not been subjected to the rigorous preclinical pharmacological testing and formal clinical trials. Moreover, the active ingredients and dosage of active medication is unknown, quality is not publicly controlled, and mechanism of action is not clear.[44] The extensively studied phytotherapeutic agent Serenoa repens (saw palmetto) has shown mild to moderate efficacy in reducing nocturia, increasing maximal urinary flow, and improving Symptom Score in men with BPH.[45]

### 

#### Saw Palmetto

Saw Palmetto, extract of the berries of the dwarf palm tree of S. repens (family Arecaceae) is most widely used.[45] The liposterolic extract contain β-sitosterol, chemically related to cholesterol, which has inhibitory effects on 5α-reductase. Various additional mechanisms have also been suggested, including inhibition of binding of DHT to cytosolic androgen receptors in prostate cells and anti-inflammatory effect. However, it has no effect on prostate volume or the prostate-specific antigen test, but slightly decreases the prostate epithelium. It does not cause impotence, but the herb may aggravate chronic gastrointestinal disease such as peptic ulcer.[46] It has been reported that oral administration of 160 mg *S. repens* twice daily for 1 to 3 months is generally superior to placebo in improving subjective and objective symptoms of BPH. ProSafe Forte is a phytochemical composition specially developed by Danor to prevent and ameliorate BPH and prostatic carcinogenesis (http://www.DanorLtd.htm).[47] Serenoa repens is currently available in France, Germany, and Spain.[48]

## Others

Novel approaches like gene therapy,[49] COX-2/LOX-5 inhibitors,[50] vitamin D _3_analogues,[51] antibody-dendrimer conjugates,[52] oxytocin antagonists,[53] and radionucleotide therapy[54] are currently exploring their role in BPH. NX-1207 has been recently announced as new treatment for the BPH.

### 

#### NX-1207

NX-1207, originally derived for treatment of Alzheimer’s disease, was later on tested for its potential role in treatment of BPH. This novel drug, developed by Nymox, is currently under Phase 3 clinical trial. It has been reported that men treated with single dose (2.5 mg dose) of NX-1207 had statistically significant improvements; the drug is administered in an office procedure that takes only a few minutes without any pain or discomfort. In addition, there were no sexual- or blood pressure-related side effects. Unlike currently approved BPH medications, NX-1207 treatment does not require the patient to take pills daily for the rest of his life (http://www.Nymox.com,).[7,55]

#### 3. Surgical treatment

Surgical interventions are considered in case of severe symptoms and complications like urinary retention, renal failure and infection that are weighed carefully against the risk and benefits of the various treatment options.

#### Invasive procedures

The gold standard for the surgical treatment was removal of obstructing tissue by open prostatectomy[56] in early 1900s, which is now replaced by transurethral resection of prostate (TURP). TURP is the hallmark of the urologist, the one against which other therapeutic measures are compared. It takes 20 to 30 minutes to resect an average gland weighing 30 g and carry the risks for complications like bleeding, infections, retrograde-ejaculation and low semen, low PSA level, and hospital stay including impotence and incontinence. Transurethral incision of the prostate (TUIP) or bladder neck incision is recommended for smaller gland weighing <25 g and has been found to be less invasive than TURP, but the long-term effectiveness in comparison with TURP is yet to be determined.[57]

#### Minimal invasive procedures (MIT)

Over the last few years, number of MIT has been established to achieve substantial improvement in the symptoms attributed to BPH. These MIT utilizes endoscopic approach to ablate the obstructing prostatic tissue.

#### Transurethral electrovaporization (TUVP)

TUVP is modification of TURP and TUIP, and utilize high electrical current to vaporize and coagulate the obstructing prostate tissue. Long-term efficiency is comparable with TURP, but number of patients has been found to experience irritative side effects.[58]

#### Transurethral microwave thermotherapy (TUMT)

More specific destruction of malignant cells without affecting normal cells can be achieved by raising the temperature of the cells using low-level radiofrequency (microwave) in the prostate up to 40 to 45^°^C (hyperthermia), 46 to 60^°^C (thermotherapy), and 61 to 75^°^C (transrectal thermal ablation).[56] TUMT has been found to be safe and cost effective, with reasonable improvement in urine flow rate and minimal impairment on sexual function.[59]

#### Transurethral needle ablation (TUNA)

It is a simple and relatively inexpensive procedure which utilizes needle to deliver high-frequency radio waves to destroy the enlarged prostatic tissue. TUNA is a successful treatment for small-sized gland and it poses a low or no risk for incontinence and impotence.[60]

#### Laser ablation

Laser prostatectomy has become an increasingly widespread form of MIT. Four types of lasers have been used to treat LUTS, namely neodymium: yttrium-aluminum-garnet (Nd: YAG) laser, holmium YAG laser (Ho:YAG), potassium titanyl phosphate (KTP), and diode laser. It has been found to be safe and effective technique, with significant improvement in urinary flow rates and symptoms. Short operative time, minimal blood loss and fluid absorption, decreased hospital stay, impotence rates, and bladder neck contractures are few of the advantages of laser prostatectomy over the TURP and other conventional techniques.[61]

#### High-intensity focused ultrasound (HIFU)

Effective protein denaturation and coagulative necrosis of prostatic tissue have been achieved by using HIFU frequencies of 4 MHZ. Significant increase in uroflow and a decrease in postvoid residual volume have been observed, but the cost is three times higher than that of TURP.[62]

#### Transurethral ethanol ablation of the prostate

Transurethral injection of absolute ethanol into the lateral lobes of prostate produces necrotic effect on prostatic tissues, leading to fibrosis and shrinkage. Significant improvement has been reported in AUA symptoms score. Continual research is going on to dilute negative factors like urinary retention, pain, dysuria, and prolonged period of catheterization with the aim to deliver safe, effective, and economical potential treatment.[63]

#### Water-induced thermotherapy

It is a simple technique that uses a cylindrical balloon to circulate hot water, resulting in even coagulation necrosis in the prostate by raising the temperature of the prostatic cells up to 60 to 70^°^C, without having major effect on nontargeted tissues.[64]

#### Plasma kinetic tissue management system (Gyrus)

Gyrus is a new technique under development and vaporizes the obstructing tissue by using plasma energy in a saline environment. Procedure has been found to be safe and effective with minimal risk of water intoxication (TURP syndrome) and generally reserved for patients on high risk.[65]

## Conclusion

BPH is the nonmalignant enlargement of the prostate gland and a common cause of voiding dysfunction in men. The primary goal of the treatment is not only to improve urinary flow and reduce symptoms scores, but also to prevent serious complications and improve quality of life. Selection of therapy depends on a number of factors like history, severity of symptoms, procedural complications, and associated side effects. Watchful waiting is more appropriate for men with mild symptoms. Safe and effective treatment with 5α-reductase inhibitors and α1 -AR antagonists can be achieved in patient with mild to moderate symptoms. Clinical efficacy of these agents has been further improved by using combination therapy; however, long-term outcomes of this study are still awaited. Traditional surgical treatment has been reported by TURP and minimally invasive techniques like hyperthermia and lasers. Current research is multimodal; many more potential new therapies with combination of old and new approaches are under development that may improve the treatment outcome.
